# Impact of Asthma and Corticosteroid Exposure on Glycemic Markers in Overweight and Obese Children

**DOI:** 10.2147/DMSO.S614951

**Published:** 2026-10-19

**Authors:** Safa Noor, Alaa Abdelhamid, Yaman Al-Haneedi, Omar Tluli, Harshita Shailesh, Elwaseila Hamdoun, Mutasim Abu-Hasan, Ibrahim A Janahi

**Affiliations:** 1 Department of Pediatric Medicine, Division of Pulmonology, Sidra Medicine, Doha, Qatar; 2 College of Medicine, Qatar University, Doha, Qatar; 3 Department of Pediatric Medicine, Division of Endocrinology and Diabetes, Sidra Medicine, Doha, Qatar; 4 Department of Pediatrics, Weill Cornell Medicine, Doha, Qatar

**Keywords:** HbA1c, corticosteroids, pediatrics, BMI, childhood asthma

## Abstract

**Purpose:**

Corticosteroids are the cornerstone of asthma management, yet they are associated with hyperglycemia and insulin resistance. This study evaluated associations between asthma characteristics and glycemic markers in children with overweight or obesity and examined whether corticosteroid exposure modifies these associations.

**Methods:**

This cross-sectional study included 199 children aged 6–17 years with overweight or obesity from the Middle East and North Africa region. They were divided into two groups: with asthma (OO-A) (N = 100) and without asthma (OO) (N = 99). Asthma phenotype was classified as type 2 (blood eosinophils ≥ 300 cells/µL, FeNO ≥ 30 ppb, or confirmed allergy) or non-type 2 asthma. Asthma severity was classified based on FEV_1_% predicted: mild (≥ 80%), moderate (60% to < 80%), and severe (< 60%). Linear and logistic regression models assessed associations between asthma characteristics and glycemic markers in the circulation before and after adjusting for corticosteroid use.

**Results:**

Asthma was associated with higher HbA1c (β = 0.152, p = 0.007) and prediabetes (HbA1c ≥ 5.7%) (OR = 2.69, p = 0.026) only when not adjusting for corticosteroid use. Mediation analysis showed that corticosteroid exposure may account for 45.4% of the total effect of asthma on HbA1c (p = 0.024). The OO group had a higher prevalence of insulin resistance (HOMA-IR ≥ 2.5) (p = 0.029). Type 2 asthma was associated with higher HbA1c without corticosteroid adjustment (β = 0.176, p = 0.002), which was attenuated after corticosteroid adjustment (β = 0.116, p = 0.057). Severe asthma remained significantly associated with higher HbA1c in both models regardless of corticosteroid adjustment (β = 0.347–0.409, p < 0.05). No consistent associations were found with other glycemic markers.

**Conclusion:**

The asthma–HbA1c association in children with overweight/obesity may be largely mediated by corticosteroid use. Severe asthma may have a direct association with HbA1c independent of steroid use. Given the cross-sectional design and limited sample sizes, these findings require confirmation in larger longitudinal studies with more precise assessments of corticosteroid exposure and other confounders to clarify causal pathways and to evaluate whether optimizing asthma management while minimizing corticosteroid exposure could reduce long-term metabolic risk in this vulnerable population.

## Introduction

Asthma is a chronic inflammatory airway disease and is considered the most common respiratory disease in children worldwide with significant respiratory related mortality and morbidity.^1^,^2^ Recent estimates from the Global Burden of Disease (GBD) study indicate that approximately 126 million children and adolescents worldwide were living with asthma in 2021, corresponding to an age-standardized prevalence of 3936 cases per 100,000 population.^3^

The airway inflammation in asthma is due to a complex interaction between genetic susceptibility and environmental exposures which leads to variable airflow limitation, bronchial hyperresponsiveness, and airway remodeling. Most therapeutic efforts in asthma are focused on targeting airway inflammation. Corticosteroids are considered the cornerstone therapy in pediatric asthma due to their potent anti-inflammatory effect, which results in controlling symptoms and preventing exacerbations.^2^,^4–6^ Inhaled corticosteroids (ICS) are the preferred long-term controller therapy for asthma, whereas systemic corticosteroids are typically reserved for acute exacerbations or severe disease.^2^ Corticosteroids exert their anti-inflammatory effects through diverse mechanisms, including inhibition of pro-inflammatory transcription factors, downregulation of cytokine expression, and modulation of immune cell function.^7^ Although inhaled corticosteroids are designed to act locally in the airway and minimize systemic exposure, a fraction of the administered dose may be systemically absorbed. The extent of systemic exposure is influenced by both the daily ICS dose and treatment duration, with higher doses generally associated with greater systemic effects.^8^ In children, dose-dependent effects on the hypothalamic-pituitary-adrenal (HPA) axis have been demonstrated, with adrenal suppression being more frequently reported at higher ICS doses.^9^ Importantly, the risk of systemic adverse effects may increase with prolonged exposure, particularly when higher doses are maintained for several months.^8^,^9^ Long-term treatment at moderate doses may also contribute to HPA-axis suppression in susceptible children.^9^ Other factors, including corticosteroid type, delivery device, inhalation technique, adherence, and concomitant use of nasal or oral corticosteroids can further influence systemic exposure.^8–10^ Importantly, systemic absorption of inhaled steroids in patients with asthma is concerning because it may lead to side effects such as glucose dysregulation.^11^

Corticosteroids may impair glucose metabolism through multiple mechanisms, including increased hepatic gluconeogenesis, reduced peripheral glucose uptake, altered pancreatic β-cell function and impaired insulin synthesis and secretion.^12^,^13^ The impact of corticosteroid therapy on glucose metabolism in children with asthma remains limited and inconsistent. A case-control study of 141 children with asthma who were treated with low-dose inhaled corticosteroids demonstrated significantly higher HbA1c levels compared to 52 healthy controls.^14^ However, more recently, a prospective cohort study published in 2024 involving 48 asthmatic children aged 2–15 years found no significant change in HbA1c after six months of continuous ICS therapy, with neither ICS dose nor treatment duration significantly associated with HbA1c levels.^15^ These discrepant findings may reflect differences in study design, duration of exposure, corticosteroid dose, or variations in underlying metabolic risk factors among participants, underscoring the need for further investigation.

Childhood obesity can further accentuate metabolic vulnerability to corticosteroid therapy. Obesity is associated with insulin resistance, dysregulated adipokine signaling, and low-grade systemic inflammation, all of which influence glucose regulation.^16^ Obesity can also alter asthma pathophysiology, leading to more severe asthma which is less responsive to corticosteroid treatment.^2^ Recent multi-omics analysis from our group has identified distinct metabolomic and lipidomic signatures differentiating asthma in normal-weight vs overweight/obese children, highlighting BMI-specific metabolic pathways including sphingolipid remodeling, TCA cycle activation, and phospholipid alterations that may contribute to the asthma pathogenesis in this population.^17^ Although several pediatric studies have examined the metabolic effects of inhaled corticosteroid exposure in children with asthma, the available evidence remains limited.^18–20^ Despite the high and increasing prevalence of obesity as a comorbidity in children with asthma, studies have not examined the metabolic effects of corticosteroid therapy specifically in overweight and obese children—a population with potentially heightened metabolic vulnerability. Additionally, the influence of asthma phenotype, disease severity, and inflammatory cytokines on glycemic outcomes has not been adequately explored. To address these gaps, this study evaluated the associations between asthma characteristics (including asthma status, phenotype, and severity) and glycemic markers, particularly in overweight and obese children, and examined whether corticosteroid exposure modifies these associations. The novelty of the present study lies in its focus on children with overweight and obesity, its inclusion of asthma phenotype, disease severity, and inflammatory cytokines, along with its assessment of multiple glycemic markers. This approach may provide a more comprehensive characterization of the metabolic effects of corticosteroid exposure in a pediatric population with potentially increased metabolic vulnerability.

## Materials and Methods

### Study Population

This study is one arm of a large, multipurpose cross-sectional study entitled “Sphingolipids in Childhood Asthma and Obesity (SOAP)”, which was conducted between August 2017 and December 2024.^21^ Participant recruitment and data collection occurred in two phases: initially at Hamad Medical Corporation (HMC) from August 2017 to May 2018, and subsequently at Sidra Medicine from October 2020 to March 2023, following a temporary pause due to institutional transition and the COVID-19 pandemic. A cohort of 199 children (aged 6 to 17 years) from the Middle East and North Africa (MENA) region who were living in the state of Qatar during that period were included in the study. The detailed description of the original study population, inclusion/exclusion criteria, and study methodology has been described in a previous publication.^21^ In addition, children with a prior diagnosis of diabetes were excluded from this study.

Study participants were divided into two groups based on their asthma diagnosis: children with overweight/obesity with asthma (OO-A) (n = 100), and children with overweight/obesity without asthma (OO) (n = 99). Asthma diagnosis was confirmed by a physician using the International Study of Asthma and Allergies in Childhood (ISAAC) guidelines.^22^ Overweight and obesity were defined as BMI equal to or greater than the 85^th^ and 95^th^ percentiles for age and sex, respectively.^23^ Children belonging to overweight/obesity with asthma (OO-A) group were recruited from the pediatric pulmonology outpatient department, whereas those belonging to overweight/obesity without asthma (OO) were recruited from the pediatric endocrinology department at Sidra Medicine, Doha, Qatar. A written informed consent was obtained from the parents of all participants. A verbal assent was obtained from all children aged 6–10 years, while a written assent was obtained from all children aged 11–18 years. The study received ethical approval from Hamad Medical Corporation (IRB No.15318/15) and Sidra Medicine (IRB No.1500770).

### Study Procedures

A detailed medical history including asthma symptoms, eczema, allergies, rhinitis, and medication use, was obtained from eligible subjects. Anthropometric measurements, including height (cm), and weight (kg) were recorded using a standard protocol. Body mass index (BMI) was calculated as weight (kg) divided by the square of height (in meters) (kg/m^2^). Furthermore, bioimpedance measurements were obtained using a body composition analyzer (Tanita BC-420MA, Tokyo, Japan) to assess parameters such as fat percentage (%), fat mass (kg), fat free mass (kg), total body water (kg), and basal metabolic rate (BMR) (kcal/day).^21^ Additionally, a physical examination was performed, including chest auscultation. Pulmonary function test (spirometry, body plethysmography, and fractional exhaled nitric oxide) measurements were conducted, as explained elsewhere.^21^

Children with asthma were classified by phenotype and stratified by disease severity. Asthma phenotype was classified as type 2 based on the presence of at least one of the following criteria: (1) blood eosinophils ≥ 300 cells/µL (0.3 × 10^9^/L), (2) FeNO ≥ 30 ppb, or (3) confirmed allergy, defined as either an allergen specific IgE level ≥ 0.35 kU/L or a positive skin prick test with a wheal diameter of ≥ 5 mm.^24–26^ Asthma was classified as non-type 2 in children who did not meet any of these criteria.^24^,^25^ Asthma severity was categorized based on forced expiratory volume in one second percent predicted (FEV_1_% predicted) as follows: (1) mild, if FEV_1_ was ≥ 80% predicted, (2) moderate, if FEV_1_ was < 80% but ≥ 60% predicted, and (3) severe, if FEV_1_ < 60% predicted.

Corticosteroid use was assessed retrospectively for all subjects by reviewing medication prescription and dispensation history as documented in their electronic medical records. Subjects were considered corticosteroid users if they were prescribed and received at least one formulation of corticosteroid medication within the last three months. Corticosteroid use was further categorized according to the route of administration as nasal, inhaled, or systemic (IV or oral). Subjects who received inhaled steroids alone or nasal plus inhaled steroids were classified as inhaled corticosteroid users. Subjects who received systemic steroids were classified as systemic corticosteroid users regardless of the concomitant use of inhaled steroids or nasal steroids. For each corticosteroid type, the following variables were extracted: (1) duration of use in the preceding three months (recorded in days), (2) time from the last steroid dose to glycemic marker measurement (recorded in days), and (3) cumulative steroid exposure. Cumulative systemic steroid doses were converted to prednisolone-equivalents in mg using standard conversion formulas.

Blood tests, including complete blood count with differential, HbA1c, 120-minutes glucose concentration, fasting plasma glucose level, and fasting insulin level were obtained in all subjects. Blood samples were collected after an 8–10 hour fasting period. HbA1c was measured using NGSP-standardized ion exchange high pressure liquid chromatography (Bio-Rad, CA, USA), 120-minutes glucose and fasting plasma glucose were assessed with a hexokinase photometric assay. Insulin levels were measured using an immunoassay (Beckman Coulter, Brea, CA, USA). The level of insulin resistance was calculated by the homeostasis model assessment of insulin resistance (HOMA-IR) using the following formula: HOMA-IR = [fasting plasma glucose (mmol/L) × fasting serum insulin (μU/mL)] / 22.5.^27^,^28^ Prediabetes was defined as an HbA1c level between 5.7% and 6.4%.^29^ In addition, 3 mL of blood was collected from each participant to measure circulating cytokine levels using a multiplex assay, as described elsewhere.^30^

### Statistical Analysis

Summary statistics are presented as mean ± standard deviation (SD) for normally distributed continuous variables, median with interquartile ranges (IQR) for skewed continuous variables, or as frequencies (%) for categorical variables. Group comparisons were performed using two-sample *t*-tests for normally distributed continuous variables and Mann–Whitney *U*-tests for non-normally distributed continuous variables, while Pearson’s chi-squared test was used to compare categorical variables between groups. Normality testing was performed using the Shapiro–Wilk test.

Linear regression analysis was used to test the adjusted associations between asthma parameters (asthma status, phenotype, and severity) and glycemic markers, generating unstandardized regression coefficients with their 95% confidence intervals (CIs). In addition, multivariable logistic regression models were employed to generate adjusted odds ratios (ORs) with 95% CIs to evaluate the association between asthma parameters and different glycemic markers. Adjusted absolute risks were also calculated from logistic regression models and are reported alongside odds ratios to facilitate interpretation. Participants with missing data were excluded from regression analyses. Directed acyclic graphs (DAGs) were used to identify confounders and determine the minimal adjustment set required for regression models (Figure S1).

Cytokines were included as covariates in the regression models based on our DAG, which identified them as common antecedents of both asthma and glycemic dysregulation rather than mediators of the asthma–glycemic marker relationship. This decision was based on evidence that immune dysregulation and systemic inflammation contribute to the pathogenesis of both asthma and impaired glucose metabolism, positioning cytokines as upstream confounders rather than intermediates on the causal pathway. Multicollinearity among cytokines was assessed using Spearman’s rank correlation coefficient. In cases where two cytokines were strongly correlated (Spearman’s ρ > 0.7) and both were deemed confounders, we retained the cytokine with stronger evidence for confounding based on prior literature and excluded the other one from our DAG to avoid multicollinearity.

The primary analysis included two separate regression models to isolate the effect of corticosteroid use from other factors as follows: Model 1 included adjustment for corticosteroid use and Model 2 did not include the adjustment for corticosteroids use. To address concerns regarding potential overadjustment for cytokines, we performed sensitivity analyses excluding cytokines from the regression models (Model 3: adjusted for corticosteroid use but not cytokines; Model 4: unadjusted for both corticosteroid use and cytokines) (Tables S2–S5). Additionally, mediation analysis was conducted where applicable to formally evaluate underlying pathways and determine whether observed associations were direct or indirectly mediated through potential intermediates. *P* values < 0.05 were considered statistically significant. All statistical analyses were performed using Stata/SE 19.5 (StataCorp, College Station, Texas, USA).

## Results

### Demographic and Clinical Characteristics

A total of 199 children were divided into two study groups: 100 children in the overweight/obese with asthma (OO-A) group and 99 in the overweight/obese without asthma (OO) group. The average age was similar between the groups (*p* = 0.41). However, the OO-A group had a higher proportion of males compared to the OO group (73.0% vs 44.4%, *p* < 0.001). Mean BMI was significantly lower in the OO-A group compared with the OO group (27.2 ± 6.0 kg/m^2^ vs 32.2 ± 8.9 kg/m^2^, *p* < 0.001). Consistently, the prevalence of obesity was higher in the OO than in the OO-A group (81.8% vs 66.0%, *p* = 0.011) (Table 1).

**Table 1:** Demographic and Clinical Characteristics of Study Participants (N = 199)

Variables	Overweight/Obesity with Asthma (OO-A) (N = 100)	Overweight/Obesity Without Asthma (OO) (N = 99)	*P* Value^†^
**Demographic Characteristics**			
Age (years)^a^	12.0 (3.4)	12.4 (2.9)	0.410
Gender, N (%)			
Male	73 (73.0)	44 (44.4)	<0.001
Female	27 (27.0)	55 (55.6)	
BMI (kg/m^2^)^a^	27.2 (6.0)	32.2 (8.9)	<0.001
Overweight/obese, N (%)			
Overweight	34 (34.0)	18 (18.2)	0.011
Obese	66 (66.0)	81 (81.8)	
**Asthma Characteristics**			
Asthma status, N (%)			
Yes	100 (100.0)	0 (0.0)	<0.001
No	0 (0.0)	99 (100.0)	
Asthma phenotype, N (%)*			
Non-type 2	12 (12.4)	–	–
Type 2	85 (87.6)	–	
Asthma severity, N (%)			–
Mild	75 (75.0)	–	
Moderate	19 (19.0)	–	
Severe	6 (6.0)	–	
**Spirometry**^‡^			
FEV_1_ % predicted^a^	89.5 (16.8)	98.9 (12.2)	<0.001
FVC % predicted^a^	98.5 (13.5)	100.1 (12.5)	0.420
FEV_1_/FVC ratio^a^	90.1 (10.2)	98.6 (6.9)	<0.001
FEF 25–75% predicted^a^	70.7 (25.6)	91.8 (20.0)	<0.001
**Clinical Characteristics**			
Eczema, N (%)	23 (23.0)	9 (9.1)	0.008
Allergies, N (%)	31 (31.0)	7 (7.1)	<0.001
Rhinitis, N (%)	51 (51.0)	3 (3.0)	<0.001
Eosinophils^b^	0.3 (0.2, 0.5)	0.2 (0.1, 0.3)	<0.001
Eosinophils (≥0.30), N (%)	54 (56.3)	29 (30.2)	<0.001
Neutrophils^b^	3.1 (2.5, 4.3)	3.7 (2.9, 4.7)	0.071
FeNO (≥ 30 ppb), N (%)	53 (55.2)	16 (18.6)	<0.001
**Corticosteroid Exposure**			
**Steroids in Last 3 Months**			
No steroids in last 3 months, N (%)	74 (74.0)	94 (94.9)	<0.001
Nasal steroids, N (%)	3 (3.0)	2 (2.0)	
Inhaled steroids, N (%)	14 (14.0)	0 (0.0)	
Systemic steroids (IV, oral and other), N (%)	9 (9.0)	3 (3.0)	
**Duration on Steroids in Last 3 Months**			
Nasal steroids (days)^b^	7.0 (3.0, 8.0)	90.0 (90.0, 90.0)	0.076
Inhaled steroids (days)^b^	87.5 (13.0, 90.0)	–	–
Systemic steroids (IV, oral and other) (days)^b^	1.0 (1.0, 1.0)	90.0 (1.0, 90.0)	0.120
**Time From Last Steroid Dose to Glycemic Marker Measurement**			
Nasal steroids (days)^b^	0.0 (0.0, 0.0)	0.0 (0.0, 0.0)	–
Inhaled steroids (days)^b^	41.5 (0.0, 60.0)	–	–
Systemic steroids (IV, oral and other) (days)^b^	13.0 (9.0, 54.0)	37.0 (0.0, 69.0)	0.710
**Cumulative Steroid Exposure in Last 3 Months (Prednisolone-Equivalents)**			
Nasal steroids (mg)^b^**	–	–	–
Inhaled steroids (mg)^b^**	–	–	–
Systemic steroids (IV, oral and other) (mg)^b^	66.7 (40.0, 156.3)	2700.0 (26.7, 7200.0)	0.640
**Glycemic Markers**			
Hemoglobin A1c (%)^a^	5.50 (0.32)	5.37 (0.33)	0.007
Hemoglobin A1c (≥5.7%), N (%)	31 (32.0)	14 (14.9)	0.005
120-minutes plasma glucose level (mmol/l)^a^	6.61 (1.46)	6.47 (1.42)	0.520
Fasting plasma glucose level (mmol/l)^a^	5.52 (0.46)	5.57 (0.40)	0.400
Fasting insulin level (μlU/l)^b^	11.99 (8.36, 21.05)	14.71 (10.87, 23.21)	0.082
Insulin resistance (HOMA-IR)^¶b^	3.58 (2.04, 5.48)	3.63 (2.53, 5.58)	0.450
HOMA-IR (≥2.50), N (%)	50 (64.1)	66 (79.5)	0.029

**Notes**: ^a^ Mean and SD; ^b^ Median (IQR); † Two sample *t*-test/Man-Whitney *U*-test/Pearson’s Chi-squared test/Wilcoxon rank-sum test; ¶ insulin resistance HOMA-IR = [fasting glucose (mmol/l) × fasting insulin (μU/ml)] / 22.5. *In the OO-A group, 3 participants (3%) had missing allergy data and did not fulfil the criteria for either blood eosinophil ≥ 300 cells/µL (0.3 × 10^9^/L) or FeNO ≥ 30 ppb, hence they could not be classified as asthma type 2 or non-type 2. **Nasal and inhaled corticosteroid doses were not available, so cumulative steroid exposure could not be calculated.

**Abbreviations**: FEV_1_, Forced Expiratory Volume in 1 Second; FVC, Forced Vital Capacity; FEF 25–75%, Forced Expiratory Flow between 25% and 75% of vital capacity; FeNO, Fractional exhaled Nitric Oxide; HOMA-IR, Homeostasis Model Assessment of Insulin Resistance.

Among children with asthma, 85 (87.6%) had a type 2 asthma phenotype and 12 (12.4%) as non-type 2. In terms of disease severity, most children had mild asthma (75.0%), followed by moderate asthma (19.0%) and severe asthma (6.0%) (Table 1). Spirometry parameters were significantly lower in the OO-A group compared to the OO group, including FEV1% predicted (*p* < 0.001), FEV1/FVC (*p* < 0.001), and FEF25-75% predicted (*p* < 0.001). No significant difference was observed between the two groups in FVC% predicted (Table 1). The proportion of children with FeNO ≥ 30 ppb was significantly higher in the OO-A group compared to the OO group (55.2% vs 18.6%, *p* < 0.001) (Table 1).

Clinical comorbidities were more frequent in OO-A group compared to the OO group, including eczema (23.0% vs 9.1%, *p* = 0.008), allergies (31.0% vs 7.1%, *p* < 0.001), and rhinitis (51.0% vs 3.0%, *p* < 0.001). Markers of eosinophilic inflammation were also elevated in the OO-A group compared to the OO group, including median eosinophil counts (0.3 vs 0.2 ×10^9^/L, *p* < 0.001) and proportion with eosinophils ≥ 0.30 (56.3% vs 30.2%, *p* < 0.001). Neutrophil levels were similar between the two groups (3.1 vs 3.7 ×10^9^/L, *p* = 0.071) (Table 1).

The majority (74.0%) of children in the OO-A group had not received any corticosteroids in the past three months. Only 14.0% received inhaled corticosteroids, 3.0% received nasal corticosteroids, and 9.0% of participants received systemic corticosteroids (oral, IV, or other). In the OO group, 94.9% of children had not used any form of corticosteroids (Table 1). Among steroid users, the median duration of inhaled corticosteroid use in the last three months in the OO-A group was 87.5 days (IQR: 13.0–90.0), while nasal and systemic steroid use durations were shorter at 7.0 days (IQR: 3.0–8.0) and 1.0 day (IQR: 1.0–1.0), respectively. The median time from the last steroid dose to glycemic marker measurement varied by steroid type: 0.0 days (IQR: 0.0–0.0) for nasal steroids, 41.5 days (IQR: 0.0–60.0) for inhaled steroids, and 13.0 days (IQR: 9.0–54.0) for systemic steroids in the OO-A group. Cumulative systemic steroid exposure (in prednisolone-equivalents) in the last three months was 66.7 mg (IQR: 40.0–156.3) in the OO-A group (Table 1).

Children in the OO-A group had significantly higher mean HbA1c and greater prevalence of prediabetes compared to the OO group (*p* = 0.007 and *p* = 0.005 respectively). No significant differences were observed between both groups in 120-minutes plasma glucose, fasting plasma glucose, or fasting insulin levels. Mean HOMA-IR did not differ significantly between both groups. However, the OO group had higher proportion of children with insulin resistance (defined as HOMA-IR ≥ 2.5) compared to the OO-A group (*p* = 0.029) (Table 1).

### Association Between Asthma Status and Glycemic Markers

There was no significant association between asthma diagnosis and HbA1c when we adjusted for corticosteroid use (Model 1). However, asthma was significantly associated with higher HbA1c levels (β = 0.152, 95% CI: 0.043–0.261, *p* = 0.007), when we did not adjust for corticosteroid use (Model 2) (Table 2). Conversely, asthma was significantly associated with higher 120-minute glucose levels in Model 1 (β = 0.585, 95% CI: 0.032–1.139, *p* = 0.038), but this association was not statistically significant in Model 2 (β = 0.421, 95% CI: −0.092 – 0.935, *p* = 0.107). No significant associations were observed for fasting plasma glucose, fasting insulin, or insulin resistance (HOMA-IR) in either model (all *p* > 0.05). In sensitivity analyses without cytokine adjustment (Table S2), these associations remained largely consistent. However, an additional association emerged: asthma became significantly associated with higher HOMA-IR in Model 3 (β = 1.639, 95% CI: 0.203–3.075, *p* = 0.026).

**Table 2:** Association Between Asthma Status and Glycemic Markers

Glycemic Marker (Sample Size)	Model 1		Model 2	
	Asthma Reg Coeff. (95% CI)	*P* Value	Asthma Reg Coeff. (95% CI)	*P* Value
**Hemoglobin A1c (%)** (N = 186)	0.093 (−0.023, 0.208)	0.117	0.152 (0.043, 0.261)	0.007
**120 minutes glucose level (mmol/l)** (N = 158)	0.585 (0.032, 1.139)	0.038	0.421 (−0.092, 0.935)	0.107
**Fasting plasma glucose level (mmol/l)** (N = 165)	−0.066 (−0.226, 0.094)	0.419	−0.109 (−0.258, 0.040)	0.151
**Fasting insulin level (μlU/l)** (N = 184)	3.267 (−1.881, 8.415)	0.212	2.373 (−2.386, 7.132)	0.326
**Insulin resistance (HOMA-IR)** (N = 158)	1.082 (−0.224, 2.389)	0.104	0.659 (−0.543, 1.861)	0.280

**Notes**: Model 1: Adjusted for age, gender, race, fat mass, steroid use, IL-10, IL-22, and TNF-α. Model 2: Adjusted for age, gender, race, fat mass, IL-10, IL-22, and TNF-α.

**Abbreviations**: CI, Confidence Interval; HOMA-IR, Homeostasis Model Assessment of Insulin Resistance.

To formally evaluate whether corticosteroid use mediates the relationship between asthma and HbA1c, we conducted mediation analysis (Table S1). The total effect of asthma on HbA1c was statistically significant (TE = 0.156, 95% CI: 0.047–0.264, *p* = 0.005). The natural indirect effect mediated through corticosteroid use was also statistically significant (NIE = 0.071, 95% CI: 0.024–0.118, *p* = 0.003), while the natural direct effect, not mediated by corticosteroids, was not significant (NDE = 0.085, 95% CI: −0.025 – 0.195, *p* = 0.130). The proportion of the total effect mediated by corticosteroid use was 45.4% (95% CI: 5.9% − 84.9%, *p* = 0.024). These findings indicate that corticosteroid use significantly mediates the observed association between asthma and elevated HbA1c in our cohort, explaining nearly half of the total effect and accounting for the attenuation of significance upon adjustment.

### Association Between Asthma Phenotypes and Glycemic Markers

Asthma non-type 2 phenotype was not significantly associated with HbA1c when we adjusted for corticosteroid use (β = 0.039, 95% CI: −0.174 – 0.252, *p* = 0.720) (Model 1) and when we did not adjust for corticosteroids use (β = 0.076, 95% CI: −0.137 – 0.289, *p* = 0.481) (Model 2). On the other hand, asthma type 2 phenotype was significantly associated with higher HbA1c levels when we did not adjust for corticosteroid use (β = 0.176, 95% CI: 0.064–0.287, *p* = 0.002) (Model 2), but this association became non-significant when we adjusted for corticosteroid use (β = 0.116, 95% CI: −0.004 – 0.236, *p* = 0.057) (Model 1). We found no significant association between either asthma phenotype and 120-minutes glucose, fasting plasma glucose, fasting insulin levels, or HOMA-IR before or after adjusting for corticosteroid use (Table 3). In sensitivity analyses without cytokine adjustment (Table S3), asthma type 2 was still significantly associated with higher HbA1c (Model 4), supporting the primary analysis. However, several new findings not observed in the primary analyses emerged. Non-type 2 asthma became significantly associated with 120-minute glucose in Model 3 (β = 1.089, 95% CI: 0.016–2.162, *p* = 0.047). Additionally, type 2 asthma became significantly associated with HbA1c (β = 0.119, 95% CI: 0.001–0.237, *p* = 0.048) and HOMA-IR (β = 1.697, 95% CI: 0.204–3.191, *p* = 0.026) in Model 3.

**Table 3:** Association Between Asthma Phenotypes and Glycemic Markers

Glycemic Marker (Sample Size)	Model 1				Model 2			
	Non-Type 2 Asthma		Type 2 Asthma		Non-Type 2 Asthma		Type 2 Asthma	
	Reg Coeff. (95% CI)	*P* Value	Reg Coeff. (95% CI)	*P* Value	Reg Coeff. (95% CI)	*P* Value	Reg Coeff. (95% CI)	*P* Value
**Hemoglobin A1c (%)** (N = 186)	0.039 (−0.174, 0.252)	0.720	0.116 (−0.004, 0.236)	0.057	0.076 (−0.137, 0.289)	0.481	0.176 (0.064, 0.287)	0.002
**120 minutes glucose level (mmol/l)** (N = 158)	1.056 (−0.033, 2.146)	0.057	0.559 (−0.016, 1.134)	0.057	0.956 (−0.130, 2.042)	0.084	0.388 (−0.137, 0.913)	0.146
**Fasting plasma glucose level (mmol/l)** (N = 165)	0.125 (−0.186, 0.437)	0.427	−0.061 (−0.226, 0.104)	0.466	0.087 (−0.222, 0.396)	0.579	−0.107 (−0.259, 0.046)	0.170
**Fasting insulin level (μlU/l)** (N = 184)	−0.866 (−10.312, 8.579)	0.857	3.121 (−2.228, 8.470)	0.251	−1.290 (−10.617, 8.037)	0.785	2.148 (−2.758, 7.054)	0.389
**Insulin resistance (HOMA-IR)** (N = 158)	0.005 (−2.465, 2.474)	0.997	1.073 (−0.285, 2.432)	0.121	−0.239 (−2.684, 2.206)	0.847	0.615 (−0.625, 1.855)	0.329

**Notes**: Model 1: Adjusted for age, gender, race, fat mass, steroid use, IL-10, IL-22, and TNF-α. Model 2: Adjusted for age, gender, race, fat mass, IL-10, IL-22, and TNF-α.

**Abbreviations**: CI, Confidence Interval; HOMA-IR, Homeostasis Model Assessment of Insulin Resistance.

### Association Between Asthma Severity and Glycemic Markers

There was no significant association between mild or moderate asthma severity and HbA1c when we adjusted for corticosteroid use (Model 1). However, severe asthma was significantly associated with higher HbA1c levels in Model 1 (β = 0.347, 95% CI: 0.038–0.655, *p* = 0.028). In Model 2 (unadjusted for corticosteroid use), mild asthma was significantly associated with higher HbA1c (β = 0.128, 95% CI: 0.013–0.243, *p* = 0.029), moderate asthma was not significantly associated with HbA1c (β = 0.173, 95% CI: −0.002 – 0.348, *p* = 0.052), but severe asthma remained significantly associated with higher HbA1c (β = 0.409, 95% CI: 0.110–0.709, *p* = 0.008). Mild asthma was also significantly associated with higher 120-minute glucose levels in Model 1 (adjusted for corticosteroid use) (β = 0.628, 95% CI: 0.035–1.220, *p* = 0.038), but this association was not significant in Model 2 (unadjusted for corticosteroid use) (β = 0.487, 95% CI: −0.062 – 1.036, *p* = 0.082). No other significant associations were observed between asthma severity and fasting plasma glucose, fasting insulin, or HOMA-IR in either model (Table 4). Sensitivity analyses without cytokine adjustment (Table S4) were largely consistent with the primary analysis. The only new finding observed was that mild asthma became significantly associated with HOMA-IR in Model 3 (β = 1.610, 95% CI: 0.091–3.128, *p* = 0.038), a finding that was not significant in the primary analysis when adjusting for cytokines.

**Table 4:** Association Between Asthma Severity and Glycemic Markers

Glycemic Marker (Sample Size)	Model 1						Model 2					
	Mild		Moderate		Severe		Mild		Moderate		Severe	
	Reg Coeff. (95% CI)	*P* Value	Reg Coeff. (95% CI)	*P* Value	Reg Coeff.(95% CI)	*P* Value	Reg Coeff. (95% CI)	*P* Value	Reg Coeff. (95% CI)	*P* Value	Reg Coeff. (95% CI)	*P* Value
**Hemoglobin A1c (%)** (N = 186)	0.068 (−0.054, 0.190)	0.272	0.126 (−0.052, 0.304)	0.165	0.347 (0.038, 0.655)	0.028	0.128 (0.013, 0.243)	0.029	0.173 (−0.002, 0.348)	0.052	0.409 (0.110, 0.709)	0.008
**120 minutes glucose level (mmol/l)** (N = 158)	0.628 (0.035, 1.220)	0.038	0.645 (−0.233, 1.524)	0.149	−0.255 (−1.799, 1.290)	0.745	0.487 (−0.062, 1.036)	0.082	0.499 (−0.361, 1.359)	0.253	−0.611 (−2.076, 0.853)	0.411
**Fasting plasma glucose level (mmol/l)** (N = 165)	−0.070 (−0.240, 0.100)	0.417	0.013 (−0.247, 0.273)	0.921	−0.313 (−0.770, 0.145)	0.179	−0.106 (−0.264, 0.051)	0.185	−0.028 (−0.282, 0.227)	0.830	−0.390 (−0.824, 0.044)	0.078
**Fasting insulin level (μlU/l)** (N = 184)	2.317 (−3.140, 7.775)	0.403	4.662 (−3.255, 12.578)	0.247	10.262 (−2.240, 22.764)	0.107	1.276 (−3.770, 6.323)	0.618	4.160 (−3.477, 11.796)	0.284	9.880 (−2.145, 21.905)	0.107
**Insulin resistance (HOMA-IR)** (N = 158)	0.911 (−0.474, 2.296)	0.196	1.912 (−0.226, 4.050)	0.079	0.696 (−2.928, 4.321)	0.705	0.477 (−0.799, 1.753)	0.461	1.638 (−0.448, 3.723)	0.123	0.274 (−3.160, 3.708)	0.875

**Notes**: Model 1: Adjusted for age, gender, race, fat mass, steroid use, IL-10, IL-22, and TNF-α. Model 2: Adjusted for age, gender, race, fat mass, IL-10, IL-22, and TNF-α.

**Abbreviations**: CI, Confidence Interval; HOMA-IR, Homeostasis Model Assessment of Insulin Resistance.

### Association Between Asthma Characteristics and Prediabetes

Asthma status, asthma phenotype, and asthma severity were not significantly associated with prediabetes when adjusted for corticosteroid use (Model 1). However, without adjustment for corticosteroid use (Model 2), children with asthma had a 2.69-fold higher odds of prediabetes compared to children without asthma (OR = 2.69, 95% CI: 1.13–6.44, *p* = 0.026). Similarly, type 2 asthma was significantly associated with higher odds of prediabetes (OR = 2.70, 95% CI: 1.13–6.45; *p* = 0.025) (Model 2). Among severity categories, only mild asthma showed a significant association with prediabetes (OR = 2.50, 95% CI: 1.01–6.19, *p* = 0.048) (Model 2). No significant associations were found between lung function parameters and the odds of prediabetes in both adjustment models (Table 5). Sensitivity analyses without cytokine adjustment were consistent with the primary findings, with asthma status, type 2 asthma, and mild asthma being significantly associated with higher odds of prediabetes in Model 4 (Table S5).

**Table 5:** Association Between Asthma Characteristics and Prediabetes

Exposure (Sample Size)	Outcome: Prediabetes (HbA1c ≥ 5.7%)					
	Model 1			Model 2		
	Odds Ratio (95% CI)	*P* Value	Adjusted Absolute Risk (%) (95% CI)	Odds Ratio (95% CI)	*P* Value	Adjusted Absolute Risk (%) (95% CI)
**Asthma Status** (N = 186)						
No	1 (Reference)	–	20.7 (11.1, 30.3)	1 (Reference)	–	15.3 (7.4, 23.1)
Yes	1.42 (0.54, 3.77)	0.476	26.0 (17.1, 34.8)	2.69 (1.13, 6.44)	0.026	31.3 (21.5, 41.0)
**Asthma Type** (N = 186)						
Non-Type 2	0.82 (0.13, 5.19)	0.836	18.9 (−2.6, 40.3)	1.36 (0.24, 7.66)	0.727	20.2 (−4.0, 44.4)
Type 2	1.37 (0.51, 3.66)	0.529	26.3 (16.7, 35.9)	2.70 (1.13, 6.45)	0.025	32.5 (21.9, 43.2)
**Asthma Severity** (N = 186)						
Mild	1.23 (0.44, 3.44)	0.689	23.6 (14.2, 33.1)	2.50 (1.01, 6.19)	0.048	29.7 (19.0, 40.4)
Moderate	2.13 (0.53, 8.60)	0.287	32.8 (11.9, 53.8)	3.29 (0.91, 11.94)	0.070	35.3 (13.1, 57.5)
Severe	2.79 (0.33, 23.80)	0.347	38.0 (−1.2, 77.2)	4.18 (0.58, 30.24)	0.156	40.5 (−0.3, 81.2)
**FEV**_**1**_ **% Predicted** (N = 183)	0.99 (0.96, 1.01)	0.301	At Mean: 47.6 (−5.0, 100.2)	0.99 (0.96, 1.01)	0.235	At Mean: 51.0 (0.7, 101.3)
**FVC % Predicted** (N = 183)	0.99 (0.96, 1.02)	0.470	At Mean: 44.0 (−19.5, 107.5)	0.99 (0.96, 1.02)	0.496	At Mean: 42.9 (−19.5, 105.2)
**FEV**_**1**_**/FVC Ratio** (N = 183)	0.98 (0.94, 1.03)	0.426	At Mean:53.4 (−29.3, 136.1)	0.97 (0.93, 1.02)	0.244	At Mean: 67.0 (−3.5, 137.4)
**FEF 25–75% Predicted** (N = 183)	1.00 (0.98, 1.01)	0.635	At Mean: 28.6 (5.7, 51.5)	0.99 (0.98, 1.01)	0.389	At Mean: 33.1 (8.6, 57.7)
**RV/TLC Ratio** (N = 170)	1.04 (0.98, 1.11)	0.154	At Mean: 12.7 (−0.3, 25.7)	1.04 (0.98, 1.10)	0.162	At Mean: 12.5 (−1.0, 26.0)
**FeNO (ppb)** (N = 170)	1.00 (0.99, 1.01)	0.746	At Mean: 23.9 (16.1, 31.6)	1.00 (0.99, 1.01)	0.496	At Mean: 22.8 (14.9, 30.8)

**Notes**: Model 1: Adjusted for age, gender, race, fat mass, steroid use, IL-10, IL-22, and TNF-α. Model 2: Adjusted for age, gender, race, fat mass, IL-10, IL-22, and TNF-α.

**Abbreviations**: HbA1c, Hemoglobin A1c; CI, Confidence Interval; FEV_1_, Forced Expiratory Volume in 1 Second; FVC, Forced Vital Capacity; FEF 25–75%, Forced Expiratory Flow between 25% and 75% of vital capacity; RV/TLC, Residual Volume to Total Lung Capacity; FeNO, Fractional exhaled Nitric Oxide.

## Discussion

In this study, we analyzed a group of 199 children who were overweight or obese and categorized into two groups: with asthma (OO-A) group and without asthma (OO) group. Our analysis showed that asthma was associated with higher HbA1c levels only in models without corticosteroid adjustment (Model 2), and this association was no longer significant when adjusting for corticosteroid use (Model 1). Mediation analysis demonstrated that corticosteroid use may significantly mediate this association, accounting for 45.4% of the total effect. When examining asthma phenotypes, type 2 asthma was significantly associated with higher HbA1c in Model 2 (without corticosteroid adjustment), but this association was non-significant in Model 1 (*p* = 0.057). Non-type 2 asthma was not significantly associated with HbA1c in either model. For asthma severity, severe asthma was significantly associated with higher HbA1c in both models, whereas mild asthma was significantly associated with higher HbA1c only in Model 2. Asthma was significantly associated with higher 120-minute glucose in Model 1 but not in Model 2; mild asthma showed a similar pattern. No significant associations were observed for fasting plasma glucose, fasting insulin, or HOMA-IR in the primary analyses. Children with asthma had significantly higher odds of prediabetes only in Model 2 (OR = 2.69, *p* = 0.026), and this association was not significant in Model 1. Similarly, type 2 asthma and mild asthma were significantly associated with higher odds of prediabetes only in Model 2. Sensitivity analyses excluding cytokines were largely consistent with the primary findings, though several additional significant associations emerged when cytokines were not adjusted for (asthma status with HOMA-IR, non-type 2 asthma with 120-minute glucose, type 2 asthma with HbA1c and HOMA-IR, and mild asthma with HOMA-IR). The attenuation of statistical significance in these associations after cytokine adjustment aligns with their role as confounders in our DAG.

Collectively, these findings may suggest that the association between asthma and glycemic dysregulation in overweight and obese children is largely mediated by corticosteroid use, although severe asthma appears to have a direct association with HbA1c that may be independent of steroid exposure and inflammation. Additionally, although type 2 asthma may confer greater metabolic risk than non-type 2 asthma, this effect was largely attenuated by corticosteroid adjustment in our cohort. However, given the cross-sectional nature of this study, modest sample sizes, and the exploratory nature of some analyses, these findings should be interpreted with caution and require confirmation in larger, adequately powered studies.

Our findings are consistent with previous studies that reported associations between corticosteroid use and elevated HbA1c or altered glucose metabolism in children with asthma. For example, a prospective observational study reported a significant increase in mean HbA1c after six months of ICS therapy suggesting that prolonged ICS therapy may affect glucose regulation in this population.^31^ Additionally, case–control data indicate that children with asthma on low-dose ICS have significantly higher HbA1c levels compared with healthy controls, reinforcing the concept that even standard anti-inflammatory therapy may influence long-term glycemic markers distinct from overt diabetes.^15^ None of the previous studies, however, examined the potential effect of comorbid obesity on the association between asthma, corticosteroids, and glycemic markers.

Interestingly, we found a higher prevalence of insulin resistance (HOMA-IR ≥ 2.50) among the OO group compared to their OO-A peers (Table 1). Although this finding may initially appear to contradict the association between asthma and high HbA1c levels in the OO-A group, it may be explained by the fact that the OO group had a significantly higher BMI compared to the OO-A group. In line with this, BMI demonstrated a positive correlation with HOMA-IR (Spearman’s ρ = 0.55, *p* < 0.0001) (Figure 1). Therefore, we postulate that the dysglycemia found in the OO group could potentially be explained by insulin resistance from excessive fat tissue as evidenced by high HOMA-IR, while the dysglycemia found in the OO-A group could be, in part, due to corticosteroid-mediated hyperglycemia as evidenced by the high HbA1c. This is consistent with well-established evidence indicating that obesity is a significant risk factor for insulin resistance in children.^32^ Together, these observations support the notion that, within our cohort, obesity-related adiposity seems to be a primary driver of insulin resistance; however, the hyperglycemia observed in children with asthma may be more closely related to factors such as corticosteroid exposure rather than persistent insulin resistance alone.

**Figure 1:**
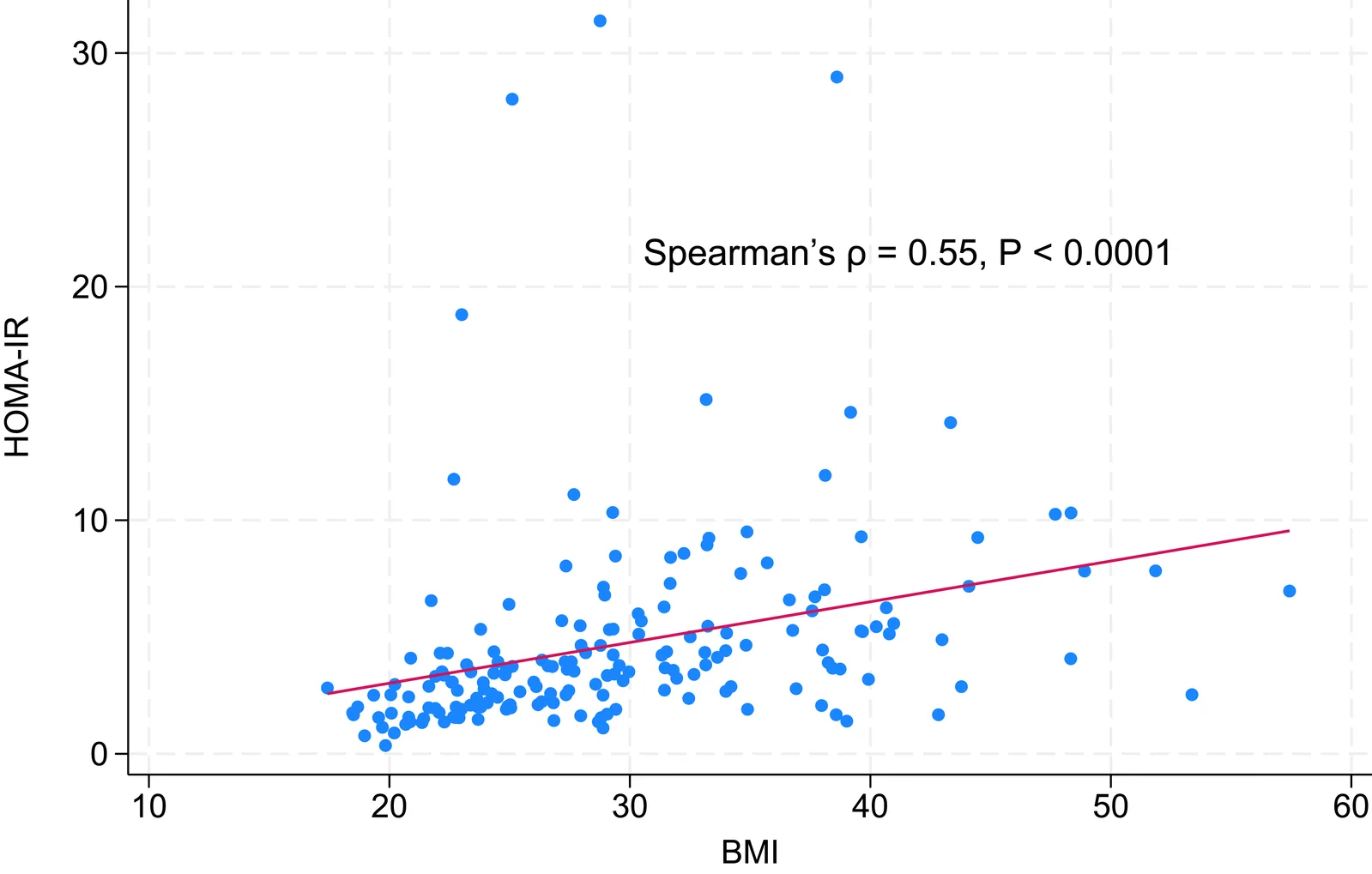
Scatter plot with line of best fit (red) for the correlation between body mass index (BMI) and homeostasis model assessment of insulin resistance (HOMA-IR).

When asthma phenotypes were examined, type 2 asthma was significantly associated with higher HbA1c levels in models without corticosteroid adjustment (Model 2: β = 0.176, *p* = 0.002), but this association became non-significant after adjusting for corticosteroid use (Model 1: β = 0.116, *p* = 0.057), whereas non-type 2 asthma was not associated with HbA1c in either model (Table 3). This pattern is biologically plausible given that type 2 asthma is characterized by eosinophilic inflammation and elevated type 2 cytokines including IL-4, IL-5 and IL-13.^33^ Although these cytokines primarily drive airway inflammation, growing evidence indicates that immune activation in the context of asthma may extend beyond the lungs contributing to systemic inflammatory processes that influence glucose metabolism.^34^,^35^ Experimental and clinical studies further demonstrate that inflammatory cytokines contribute to islet cell dysfunction and are associated with progression towards type 2 diabetes mellitus.^36^ Proposed mechanisms underlying this process include disruption of glucose homeostasis through increased hepatic glucose production, and altered adipose tissue function, all of which promote chronic hyperglycemia and metabolic dysregulation.^36^ However, our adjustment for cytokines in the primary analysis (Models 1 and 2) indicates that the type 2 asthma–HbA1c association was likely not driven by inflammation. The attenuation of statistical significance in the type 2 asthma–HbA1c association after adjusting for corticosteroid use in our cohort suggests that the observed effect may be largely mediated by steroid exposure rather than by the inflammatory phenotype itself. This is consistent with our mediation analysis, which demonstrated that corticosteroid use may mediate approximately 45% of the total effect of asthma on HbA1c. Notably, the absence of significant associations between asthma phenotypes and 120-minutes glucose, fasting glucose, insulin, or HOMA-IR further supports the notion that HbA1c is the best glycemic marker to monitor long-term glycemic changes in overweight and obese children with type 2 asthma, particularly those on corticosteroids.

A notable finding of this study was that severe asthma remained significantly associated with higher HbA1c levels even after adjusting for corticosteroid use and inflammatory cytokines (Model 1: β = 0.347, *p* = 0.028). This pattern contrasted with mild asthma, where the association with HbA1c was only significant in models without corticosteroid adjustment, suggesting that the observed effect in milder disease is largely driven by steroid exposure rather than the disease itself. The persistence of the association in severe asthma, despite adjustment for corticosteroids and cytokines, may suggest that severe disease has a direct effect on glycemic control independent of these factors. However, the small number of participants within the severe asthma subgroup (N = 6) necessitates interpretation of this finding as exploratory and hypothesis-generating, with confirmation required in larger studies.

This study has several notable strengths. First, it utilizes a well-characterized pediatric cohort with overweight/obesity which is at a heightened risk for glycemic dysregulation. Asthma status, phenotype, and severity, were all rigorously defined using established and clinically validated guidelines, ensuring standardized classification across participants and enhancing the clinical validity of our findings. Moreover, incorporating a comprehensive evaluation of glycemic markers allowed a multidimensional assessment of glucose metabolism rather than relying on only HbA1c as a measure of glycemic dysregulation. Methodologically, the use of DAGs, multivariable regression models and mediation analysis while adjusting for key covariates including measures of adiposity, inflammatory cytokines, and corticosteroid use allowed for the examination of potential confounding and mediating pathways.

Nevertheless, there are several limitations that should be acknowledged. For instance, the use of a cross-sectional design precludes us from making inferences about the causal and temporal relationships between asthma, corticosteroid exposure, and glycemic changes. The use of a dataset which primarily includes pediatric patients from the MENA region restricts the generalizability of our results to more ethnically and genetically heterogenous populations. Corticosteroid use by study subjects was obtained from their medical record. Participants were classified as corticosteroid users only if there is documentation that they had been prescribed and received at least one corticosteroid formulation within the preceding three months. Consequently, corticosteroids prescribed before this period or outside our institution may not have been captured, potentially resulting in an underestimation of corticosteroid users. This may explain the relatively low proportion of corticosteroid users in our asthma population. Nevertheless, it is unlikely to impact the overall conclusions, as significant differences in the outcomes between asthma and control groups were observed despite the low proportion of steroid users. Importantly, the lack of data on cumulative corticosteroid exposure, inhaled corticosteroid dose category, and medication adherence limits our ability to draw conclusions about the effect of corticosteroid exposure on glycemic markers or examine dose-response relationships. Additionally, despite adjusting for several covariates, residual confounding due to unavailable parameters like cumulative corticosteroid exposure, physical activity, dietary intake, socioeconomic variables, and pubertal status cannot be excluded. Despite constructing a DAG to determine the minimal adjustment set and assessing multicollinearity to avoid redundant covariates, the logistic regression models for prediabetes include many covariates relative to the number of outcome events, raising the potential for overfitting. Moreover, the wide confidence intervals in the logistic regression estimates reflect imprecision, likely due to the limited sample size. These factors warrant cautious interpretation of these findings, and larger confirmatory studies are needed. Finally, the small number of participants within the severe asthma (N = 6) and non-type 2 asthma (N = 12) subgroups renders these analyses underpowered to detect significant associations with glycemic markers and prediabetes. Estimates for these subgroups are likely unstable, potentially sensitive to outliers, and should therefore be interpreted as exploratory. These limitations, which are commonly encountered in cross-sectional study designs, underscore the need for larger, longitudinal studies.

## Conclusion

In conclusion, this study demonstrates that the association between asthma and elevated HbA1c in overweight and obese children may be largely explained by corticosteroid use, which mediated approximately 45% of the total effect. While type 2 asthma was associated with elevated HbA1c, this effect was attenuated after adjusting for corticosteroids, suggesting that corticosteroid exposure, rather than the inflammatory phenotype itself, may be the primary driver of glycemic changes in this population. However, severe asthma remained independently associated with higher HbA1c even after adjusting for corticosteroids and cytokines, suggesting a potential direct association between severe disease and glycemic control. No consistent associations were found with other glycemic markers, highlighting HbA1c as a potentially useful marker for monitoring metabolic risk in overweight and obese asthmatic children. These findings may suggest the need for metabolic surveillance in children with asthma, particularly those with severe disease or on corticosteroid therapy. However, given the cross-sectional design, limited sample sizes, and exploratory nature of some analyses, these findings suggest possible associations that require confirmation in larger, longitudinal studies with more precise assessments of corticosteroid exposure, asthma control, pubertal status, socioeconomic, and lifestyle factors. Future studies are also needed to clarify causal pathways and to determine whether optimizing asthma management while minimizing corticosteroid exposure may reduce long-term metabolic risk in this vulnerable population.

## Data Availability

The database generated and/or analyzed are available from the corresponding author upon reasonable request.

## Abbreviations

MENA, Middle East and North Africa; OO-A, Overweight/Obesity with Asthma; OO, Overweight/Obesity without Asthma; HbA1c, Hemoglobin A1c; HOMA-IR, Homeostatic Model Assessment of Insulin Resistance; ICS, Inhaled Corticosteroids; SOAP, Sphingolipids in Childhood Asthma and Obesity; ISAAC, International Study of Asthma and Allergies in Childhood; IRB, Institutional Review Board; FeNO, Fractional exhaled Nitric Oxide; FEV_1_, Forced Expiratory Volume in 1 Second; BMI, Body Mass Index; SD, Standard Deviation; IQR, Interquartile Range; ORs, Odds Ratios; CIs, Confidence Intervals.
